# Fungicide Tebuconazole Influences the Structure of Human Serum Albumin Molecule

**DOI:** 10.3390/molecules24173190

**Published:** 2019-09-02

**Authors:** Katarína Želonková, Samuel Havadej, Valéria Verebová, Beáta Holečková, Jozef Uličný, Jana Staničová

**Affiliations:** 1 Faculty of Science, Pavol Jozef Šafárik University, Jesenná 5, 041 54 Košice, Slovakia (K.Z.) (S.H.) (J.U.); 2 Department of Chemistry, Biochemistry & Biophysics, University of Veterinary Medicine & Pharmacy, Komenského 73, 041 81 Košice, Slovakia; 3 Department of Biology & Genetics, University of Veterinary Medicine & Pharmacy, Komenského 73, 041 81 Košice, Slovakia; 4 First Faculty of Medicine, Charles University, Kateřinská 1, 121 08 Prague, Czech Republic

**Keywords:** tebuconazole, interaction, human serum albumin, spectroscopy, molecular modeling

## Abstract

Studies of interactions between pesticides and target mammalian proteins are important steps toward understanding the pesticide′s toxicity. Using calorimetric and spectroscopic methods, the interaction between triazole fungicide tebuconazole and human serum albumin has been investigated. The spectroscopic techniques showed that fluorescence quenching of human serum albumin by tebuconazole was the result of the formation of tebuconazole/human serum albumin complex with the static type as the dominant mechanism. The association constant was found to be 8.51 × 10^3^ L/mol. The thermodynamic parameters were obtained as ΔH = −56.964 kJ/mol, ΔS = −115.98 J/mol·K. The main active interactions forming the tebuconazole/human serum albumin complex were identified as the interplay between hydrogen bonds and/or van der Waals forces, based on thermodynamic experiments. These binding modes were corroborated well by the predictions of molecular modeling. Hydrogen bonding of tebuconazole with Arg222, Ala215 and Ala291 of human serum albumin played a relevant role in binding. The conformation changes in secondary structure were characterized by circular dichroism and 3D fluorescence spectra.

## 1. Introduction

Pesticides are globally used for crop control. The industrial and agricultural means of food production are expected to increase, in particular, the quantity and quality of crops. On the other hand, the question arises whether using pesticides endangers the health of consumers. Knowledge about the effects and consequences of pesticide formulations′ side-effects is necessary to prevent the use of inappropriate products or to limit the extent of their use. This knowledge also makes it possible to determine pesticide tolerances and their residual limits, especially in food and drink.

**Figure 1:**
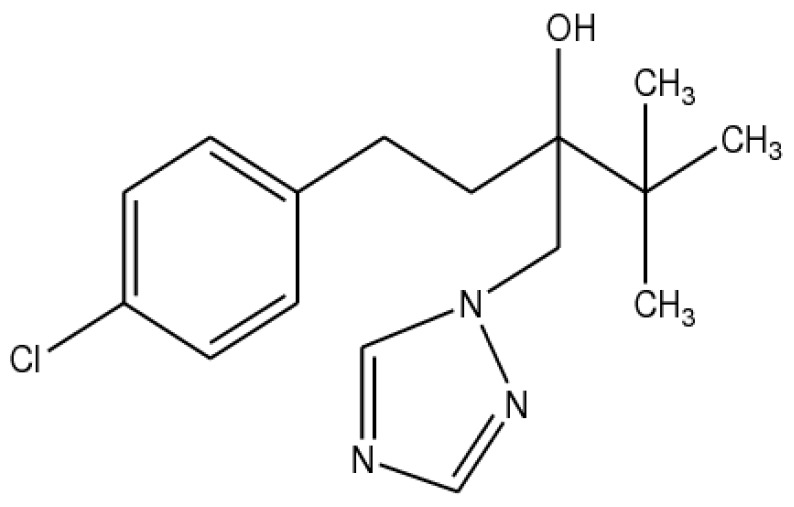
Structure of tebuconazole (TB).

Tebuconazole [1-(4-chlorophenyl)-4,4-dimethyl-3-(1,2,4-triazol-1-ylmethyl)pentan-3-ol] (Figure 1) is a representative of the triazole class of fungicides, which are used in agriculture for disease control of cereal, vegetable, fruit and nut crops worldwide [1]. The antifungal activity of triazoles is due to their ability to inhibit the ergosterol biosynthesis pathway via inhibition of enzyme 14-α-demethylase, which blocks the conversion of lanosterol to ergosterol and results in the arrest of fungal growth [2].

Tebuconazole (TB) is the active ingredient used in Orius 25 EW, which is applied to a number of crops such as fruits, rice, vegetables and grapes, because of its broad-spectrum antifungal activity. The US EPA has classified TB as Group C-Possible Human Carcinogen [3]. TB has a half-life in soil of 49–610 days under aerobic conditions and exhibits moderate mobility [4,5]. It is classified as toxic to aquatic organisms [6] and its content in stream water has increased in recent years [7]. TB concentrations detected in surface waters were up to 175–200 μg/L [8]. Yu and co-workers [9] have observed that exposure to TB can alter thyroid hormone levels as well as gene transcription in zebrafish larvae. The results obtained by Holečková et al. [10] indicated that TB can exert either cytotoxic and/or genotoxic effect on bovine peripheral lymphocytes. TB has been also found in farm workers with maximum concentrations of 19.2 μg/L and 2.22 ng/kg in urine and hair samples, respectively [11,12].

Mas et al. [13] synthesized pH-sensitive gated mesoporous silica nanoparticles loaded with TB. Interestingly, these nanodevices loaded with TB significantly enhanced TB cytotoxicity. The increase of TB action could potentially overcome the adverse side effects associated with topical therapies for vulvovaginal infection and significantly improve its cost-effectiveness.

Human serum albumin (HSA) is the most abundant protein in human plasma (~600 µM) with a typical concentration of 40 mg/mL in the bloodstream [14,15]. This protein contributes to a significant number of transport and regulatory processes and binds a wide variety of substrates such as amino acids, fatty acids, hormones, metals, and a broad spectrum of drugs [16,17,18,19,20,21]. HSA is a monomeric protein consisted of 585 amino acids with total molecular weight of 66,400 Da. HAS macromolecule is organized in three main domains (I, II, III), where each can be divided into two subdomains (A and B) containing 6 and 4 alpha-helixes, respectively.

Recently, several studies have been realized to examine the toxic effects of pesticides at the protein level [22,23,24,25], but information about the influence of triazoles on plasma proteins is still limited [26,27]. Binding of triazoles to plasma proteins has toxicological importance as it can significantly affect their distribution in and excretion from an organism [27]. Their penetration into the blood system can cause their binding to plasma proteins and afterwards induce some structure and function alteration in the protein.

The comprehensive study by Zhang et al. [26] explored the mechanism of interaction of the typical triazole fungicides with HSA using multispectroscopic techniques. They excluded determination of the binding properties of TB because of insufficient quenching of protein by TB. However, our preliminary calorimetric and spectroscopic studies [28] show significant effects of TB on HSA (fatty acid-free) thermal stability, absorption spectrum, and fluorescence quenching of protein by TB, suggesting that HSA is an important component of TB distribution. Thus, we repeated and extended our preliminary results including other spectroscopic techniques (far-UV circular dichroism spectroscopy, 3D fluorescence spectroscopy and site markers competitive experiments) to confirm and refine the TB influence on HSA structure. Molecular modeling was done with molecular docking to complement our experimental findings and to suggest their interpretation in molecular interaction terms.

In this study we complete the information about interactions of TB in relation to plasma protein (HSA) with regard to the above-mentioned preliminary results. This investigation might be beneficial in cases of frequent application of TB as pesticide. Moreover, our results may provide data required for clarifying the binding mechanisms of TB with HSA and might also be helpful for food safety and human health protection when TB is applied as an antifungal agent.

## 2. Results and Discussion

### 2.1. Differential Scanning Calorimetry

Thermal stability study of HSA, as well as investigation of the influence of ligand/HSA complex formation on this stability, can provide an initial view of the possible interaction between the protein and the fungicide. It is known, that ligand binding gives an increment in the thermal stability of protein due only to the coupling of binding with unfolding [29]. Faroongsarng [30] studied assessment of the dissociation energies of diazepam and ibuprofen bound on HSA by DSC method and found that the denaturation of HSA incubated with the drugs was done at higher temperatures than HSA itself depending on drugs′ concentrations. Similar findings were demonstrated by other authors [31,32]. The thermograms of free HSA and HSA (fixed concentrations) in the presence of different TB concentrations are shown in Figure S1. The Table 1 summarizes thermodynamic parameters obtained from the thermograms in Figure S1.

A slight shift in HSA denaturation temperature (more than 3 °C) due to interaction with TB can be deduced from Table 1. We also found complete irreversibility of the denaturing process. This irreversibility of HSA denaturation was confirmed in all samples, which agrees very well with other published results [33]. The DSC curves were fitted to a non-two-state model, because this model provided the best fit results. Increase in temperature and enthalpy of denaturation (Table 1) is compatible with the presence of high affinity sites in the folded conformation of proteins [29]. Unlike the above-mentioned thermostability, a decrease in thermodynamic parameters means the existence of multiple low-affinity binding sites in the unfolded conformations of HSA [34]. We can assume that the native structure of HSA is affected by TB molecules, because thermostability signals stabilizing of the TB/HSA complex formation. However, the increasing thermodynamic parameters (denaturation temperature and enthalpy) measured at one scanning rate do not give clear information about the TB/HSA thermostability. Lemli et al. [35] measured thermodynamic parameters including activation energies of BSA in absence and presence of tilmicosin on depending on three different scan rates and found the decrease of thermodynamic parameters at two scan rates. They concluded that the ligand tilmicosin promotes the thermal denaturation of the protein. As we did not perform our measurements at different scan rates we cannot significantly declare on the binding of TB with protein.

### 2.2. Fluorescence Quenching

Fluorescence spectroscopy is a very frequently used method to investigate the interactions between small molecules and macromolecules including proteins. Prior to fluorescence experiments the UV-VIS absorption spectra of TB (Figure S2), HSA, and TB/HSA complex were taken (not shown). The UV-VIS absorption spectrum of TB shows two strong peaks at 208 and 220 nm measured at maximal used TB concentration (Figure S2). On the contrary, well-known UV-VIS absorption spectrum of HSA is characterized by the strong peak at 210 nm and the weak peak at 280 nm, which is due to the aromatic acids. Localization of the aromatic acids in the binding sites of HSA macromolecule led to use the wavelength 280 nm as an excitation wavelength in fluorescence quenching measurements. To separate fluorescence spectra of Tyr and Trp, we used the excitation wavelength 295 nm.

**Figure 2:**
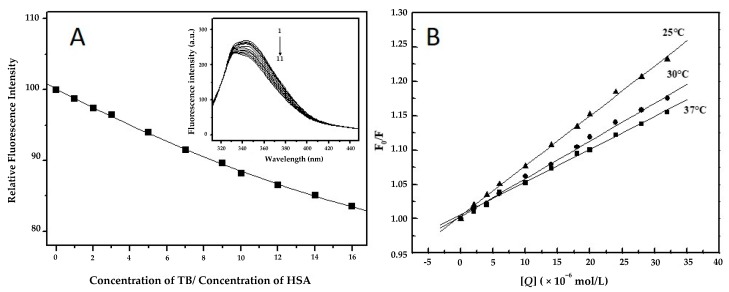
(**A**) Decrease of HSA fluorescence in the absence and presence of TB. Inset: Fluorescence spectra of TB/HSA complexes; c (HSA) = 2 × 10^−6^ mol/L, c (TB) = 0–32 × 10^−6^ mol/L (1–11); λ_exc_ = 295 nm; λ_em_ = 300–500 nm; pH = 7.4; t = 25 °C [28]. (**B**) Stern-Volmer plots for the binding of TB with HSA at different temperatures.

We repeated our preliminary fluorescence quenching experiments at room temperature [28] to confirm and specify the interaction between TB and HSA. The decrease of HSA fluorescence in the absence and presence of TB measured at 25 °C is shown in Figure 2A. From this figure it can be seen that the intensity of fluorescence of HSA declines with the raising concentration of pesticide, which is due to fluorescence quenching. We observed no shift in maximum emission wavelength of HSA (Figure 2A inset), suggesting that small molecules are likely to interact with HSA via the hydrophobic region located inside the protein [26].

The values of *K_SV_*, *K_A_* and *n* at 25 °C have been already published by our group [28]. We realized additional measurements at 30 °C and 37 °C to find a temperature dependence of fluorescence quenching [36].

Fluorescence quenching can be induced by processes such as ground state complex formation, excited-state reactions, energy transfers and molecule rearrangements [36]. Generally, the quenching mechanisms are divided as static quenching and dynamic quenching, which can be characterized by their different dependence on temperature [37]. By dynamic quenching, the quenching constants of the fluorescent complexes increase with rising temperature, because dynamic quenching depends primarily on diffusion. On the contrary, by the static quenching, the increase in temperature results in decreased stability of complexes; the values of quenching constants will be lower. The consequence of higher temperature is faster diffusion and broader amounts of collisional quenching and this typically leads to the dissociation of weakly bound complexes [38].

The possible quenching mechanism can be interpreted with the Stern-Volmer Equation (1) [36,39]:

(1) $$\frac{F_{0}}{F}=1+K_{q}\tau_{0}\left[Q\right]=1+K_{SV}\left[Q\right]$$

where *F_0_* and *F* are the intensities of fluorescence of the biomacromolecule before and after addition of quencher (TB), respectively. *K_q_* is the quenching rate constant of the biomacromolecule, $\tau_{0}$ is the average lifetime of the fluorescence of biomacromolecule without presence of quencher (about 10 ns for most of the fluorophores [40], [*Q*] is the concentration of quencher (TB), and *K_SV_* is the Stern-Volmer constant. Figure 2B shows Stern-Volmer plots for fluorescence quenching of the complex TB/HSA at three different temperatures. The corresponding *K_SV_* is summarized in Table 2. The value of *K_SV_* is inversely correlated with the temperature, implying that the quenching mechanism of HSA by TB is initiated by static quenching.

### 2.3. Determination of Binding Parameters

The binding parameters such as number of binding sites (*n*) and the association constant (*K_A_*) can be estimated from the Hill Equation (2) [41,42,43]:

(2) $$\frac{\log\left(F_{0}-F\right)}{F}=\log K_{A}+n\log\left[Q\right]$$

where F_0_ and F are the intensities of fluorescence of biomacromolecule before and after addition of quencher (TB) and [*Q*] is the total quencher concentration. By plotting log (*F_0_* − *F*)/*F* versus log [*Q*], the association constant and number of binding sites *n* can be obtained. Hill plots of TB quenching effect on fluorescence of HSA at the different temperatures are displayed in Figure S3.

The corresponding *K_A_* and *n* are summarized in Table 2. The results show a slight binding affinity between HSA and TB. The number of binding sites *n* in HSA approximates to one signaling that only one site is reactive to TB in the experimental concentration range.

In comparison, the interaction of four other triazole fungicides (triadimefon, imazalil myclobutanil, penconazole) with HSA has been reported by Zhang et al. [26]. The association constants determined by means of fluorescence quenching in their study range from 3.96 × 10^3^ L/mol (triadimefon) to 8.47 × 10^3^ L/mol (penconazole) [26]. Our association constant for TB/HSA complex (8.51 × 10^3^ L/mol) approaches those constants very well. The number of binding sites for TB, which is 1.01 ± 0.02 (Table 2) corresponds with those obtained by Zhang et al. [26]. Considering the chemical structure of all triazole fungicides, we may assume that both TB and penconazole molecules look similar. This fact can be one of several other reasons explaining their very similar binding affinity expressed in terms of association constants. For comparison, the binding affinities of other pesticides to HSA are reported in the studies by Wang [24,25]. They used the spectroscopic approach to find association constants for imidacloprid/HSA and thiacloprid/HSA complexes respectively. Both molecules represent neonicotinoid insecticides currently used in agriculture. Comparing the association constants of triazole and neonicotinoide pesticides, we can declare that the binding affinity to HSA of triazole fungicides including TB to HSA is lower than that of neonicotinoides. However, the differences are not very pronounced.

### 2.4. Thermodynamic Parameters

Interactions between small molecules and biomacromolecules include four types of non-covalent interactions, hydrophobic, hydrogen, van der Waals forces and electrostatic forces. Thermodynamic parameters such as Gibbs free energy change (Δ*G*), entropy change (Δ*S*) and enthalpy change (Δ*H*) can give an indication useful for defining interaction modes [43]. If Δ*H* does not change significantly over the temperature range studied, it can be regarded as a constant, and then Δ*H*, Δ*G* and Δ*S* are estimated from the van’t Hoff Equation (3):

(3) $$\ln K_{A}\;=\;\frac{-\Delta H}{RT}\;+\;\frac{\Delta S}{R}$$

where *K_A_* is the association constant at temperature *T* and *R* is the universal gas constant (*R* = 8.314472 J/K·mol). Van′t Hoff plot for the interaction TB and HSA is shown in Figure S4.

The Gibbs free energy change can be obtained from the Equation (4):

(4) ΔG=ΔH−TΔS

Previous studies [44,45] have defined the magnitudes and signs of the thermodynamic parameters correlated with various individual kinds of interaction. For Δ*H* < 0 and Δ*S* > 0, the hydrophobic interactions play major role, for Δ*H* < 0 and Δ*S* < 0, van der Waals forces and hydrogen bond formation are suggested as more important, whereas for Δ*H* ≈ 0 and Δ*S* > 0, contributions to these changes are associated with electrostatic forces [46]. The calculated values of Δ*H*, Δ*G* and Δ*S* are summarized in Table 2. The negative values of Δ*G* indicate the spontaneous binding process. The values of Δ*H* and Δ*S* are negative, which indicates that the van der Waals forces or/and hydrogen bonds play a main role in binding process. We assume that this situation is caused by the existence of the OH group in TB, which is the hydrogen bond donor. However, N atoms, which are located on the triazole ring can very easily form hydrogen bonds with amino acid residues such as Arg and tryptophan (Trp). Besides, TB contains aromatic rings, and therefore π→π stacking interactions with amino acid residues such as His, Tyr and Trp, in HSA are also reasonable.

### 2.5. Synchronous Fluorescence Spectroscopy

Synchronous fluorescence spectroscopy involves the simultaneous scanning of excitation and the fluorescence monochromators of a fluorimeter, while maintaining a settled wavelength difference (Δλ, D-values) between them. Synchronous fluorescence spectra are used to study the molecular environment in the vicinity of a fluorophore. The advantages of this method are spectrum simplification, spectral band reduction and prevention of various perturbations [47]. Vekshin [48] suggested a useful method to study the environment of amino acid residues by measuring the possible shift in wavelength emission maximum, especially the shift position of the emission maximum corresponding to changes in polarity around the chromophore molecule.

The emission spectra of HSA come from three kinds of fluorophores, namely Phe, Tyr and Trp, whose fluorescence spectra overlap. When the D-values (Δλ) between excitation wavelengths and emission wavelengths were settled at 15 nm and 60 nm, the spectra provided the individual information for amino acids residues Tyr and Trp in the protein, respectively [49]. Several authors [16,50,51] found that in the HSA molecule structure the subdomain IIA contains a large hydrophobic cavity, which prefers to accept bulky heterocyclic anions like TB is. Based on these findings, it can be supposed that the primary binding site of triazole fungicides including TB is likely to be subdomain IIA where Trp214 is only Trp in the HSA molecule.

The fluorescence spectra characteristic of Tyr and Trp residues are shown in Figure S5. It is obvious that the intensities of the Trp residues are stronger than those of Tyr residues. The maximum emission wavelengths remain unchanged during the interaction, suggesting that polarity around amino acids residues Tyr and Trp is unchanged. No significant change was found in the fluorescent emission peak position of both Tyr and Trp residues. Thus, the addition of TB did not rearrange the microenvironment of Tyr and Trp in HSA.

### 2.6. Site Markers Competitive Experiments

To elucidate the binding site of TB to HSA, we made the competitive experiments. The measurements were carried out in the presence of two ligands ketoprofen (KTF) and ibuprofen (IBF). KTF and IBF, nonsteroidal anti-inflammatory drugs, have been identified as stereotypical ligands for Sudlow′s sites I and II (subdomain IIA and IIIA), respectively [52].

**Figure 3:**
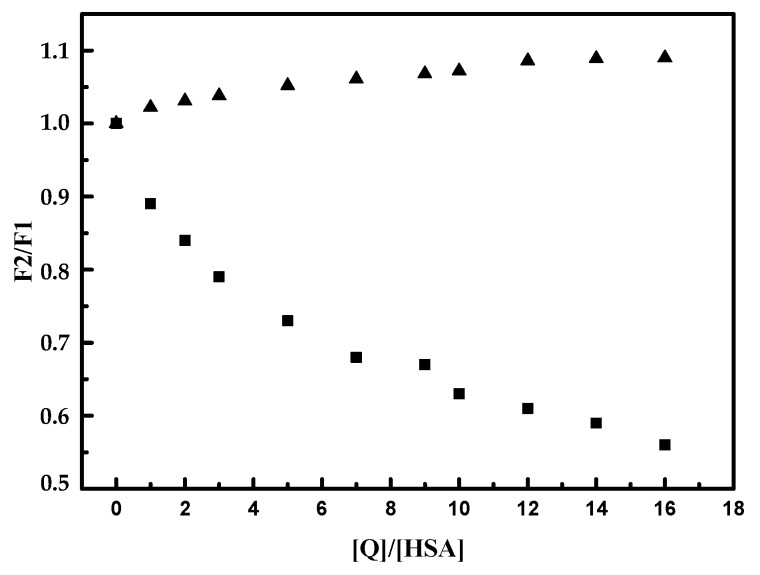
Effect of ketoprofen (KTF) and ibuprofen (IBF) on fluorescence intensity of the TB/HSA system; c (TB) = c (HSA) = 2 × 10^−6^ mol/L; c(KTF) = c(IBF) = 0–32 × 10^−6^ mol/L; [*Q*]: ▲ ibuprofen ■ ketoprofen; pH = 7.4; t = 25 °C.

With the addition of TB to the KTF/HSA and IBF/HSA mixtures, the intensity of fluorescence of HSA gradually decreased (Figures S6 and S7). To comparison of the influence of KTF and IBF on the binding TB to HSA, the association constants in the presence of site markers were calculate from Hill Equation (2) (Table S1). The results show that in the presence of KTF, the association constant of TB/HSA complex was very low in comparison to this case when IBF was present in the mixture. So the presence of IBF in the mixture caused only a small change in the binding parameters (Table S1). These results indicate that the bound of TB to HSA was affected by the presence of KTF, while IBF did not prevent the interaction of TB in its usual location. This analysis indicated that the binding site of TB was mainly located within Sudlow site I (subdomain IIA) of HSA. To verify above mentioned results, we used the method originated from Sudlow et al. [53]. This method dealt with probe (KTF, IBF) displacement, which can be expressed by following Equation (5):

Probe displacement = *F_2_/F_1_* (5)

where *F_2_* represents the fluorescence intensity of TB/HSA system in the presence of the probe and *F_1_* represents the fluorescence intensity of TB/HSA system in the absence of the probe. As shown on Figure 3 the increasing concentration of ketoprofen led to decreasing of fluorescence intensity of TB/HSA complex. However, the increasing concentration of ibuprofen has a little impact on fluorescence intensity of TB/HSA complex. The displacement of TB bound to HSA after addition of KTF indicated that site I (subdomain IIA) was the probable binding site of TB to HSA.

### 2.7. 3D Fluorescence Spectroscopy

**Figure 4:**
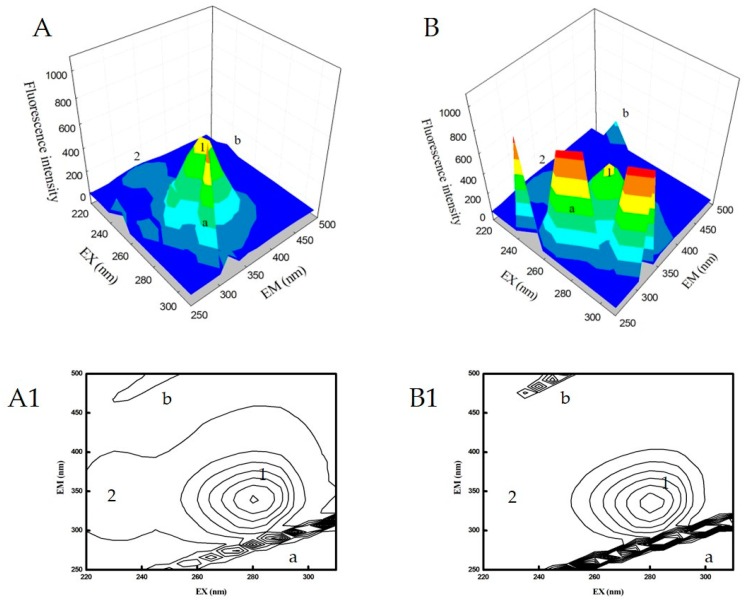
3D fluorescence spectra of HSA in the absence (**A**, **A1**) and in the presence of TB (**B**, **B1**). c (HSA) = 2 × 10^−6^ mol/L; c (TB) = 32 × 10^−6^ mol/L; pH = 7.4; t = 25 °C.

To analyze the changes in protein secondary structure after interaction with TB, 3D fluorescence spectroscopy was utilized. 3D fluorescence spectroscopy has been commonly used for studying the interaction between proteins and small molecules in recent years. The fluorescence intensity, emission wavelength and excitation wavelength are the parameters used to investigate the synthetic formation of the samples. In addition, the contour fluorescence spectra can also provide some meaningful information [54,55]. The 3D fluorescence spectra and the contour fluorescence spectra for HSA and TB/HSA are displayed in Figure 4.

Peak a (λ_ex_ = λ_em_) is known as the Rayleigh scattering peak, and peak b (2λ_ex_ = λ_em_) is known as the second-order scattering peak, and they arise from elastic light scattering on particles smaller than the wavelength of light. The intensity of both scattering peaks increases with addition of TB resulting in an increase in the diameter of the molecule and thus greater scattering [55,56]. Peak 1 (λ_ex_ = 275 nm, λ_em_ = 340 nm) represents the intensity of fluorescence of Tyr and Trp amino acid residues involving π→π* transition, reflecting some changes in the tertiary structure. Peak 2 (λ_ex_= 230 nm, λ_em_= 330 nm) is mainly caused by n→π* transition of protein polypeptide backbone structures and signifies changes in the secondary structure of HSA [26,57].

From the figures it can be seen that the fluorescence emission intensity of peak 1 decreased upon the addition of TB. Similarly, the fluorescence of peak 2 also decreased, which implies that interaction with TB possibly caused some minor changes in stabilization of HSA and a slight unfolding of its polypeptide backbone, resulting in conformational changes. The intensities of the measured peaks are summarized in Table S2.

### 2.8. Circular Dichroism

To collect more information on the binding of TB to HSA, circular dichroism spectroscopy was applied to investigation the secondary structure of HSA and TB/HSA complex. CD is a sensitive technique for monitoring conformational changes in proteins. HSA presents two negative bands in the ultraviolet region at 208 and 222 nm, characteristic for the α-helical structure [58,59]. These two peaks contribute to π→π* transfer (208 nm) and *n*→π* transfer (222 nm) for the peptide bond in the α-helix [60]. CD measurements performed in the absence and in the presence of different concentrations of TB displayed that the binding of TB to HSA caused a slight decrease in both bands (Figure S8). The CD results were expressed in terms of mean residue ellipticity (MRE), according to the following Equation (6):

(6) $$\mathrm{MRE}=\frac{\mathrm{Observed}\;\mathrm{CD}\left(\mathrm{mdeg}\right)}{10cnl}$$

where *c* is the concentration of HSA, *n* is the number of amino acid residues of HSA and *l* is the path length.

The α-helix contents of free HSA and TB/HSA complexes were calculated from MRE values at 208 nm using the following Equation (7) [61]:

(7) $$\alpha -\mathrm{helix}\;\left(\%\right)\;=\;\frac{-{\mathrm{MRE}}_{208\;\mathrm{nm}}-4000}{33000-4000}\;\times 100$$

Free HSA contains 55.75% α-helical structures, and in the presence of TB the content of α-helical structures gradually decreases to 53.23% at a molar ratio of 5/1. We also used ethanol to probe its effect on secondary structure of HSA. The effect of ethanol was less than 1%, so we can state that TB causes slight changes in the secondary structure of HSA.

This result shows that TB interacts with the amino acid residues of the polypeptide backbone in HSA, disrupts the hydrogen bonds [26], and evokes protein destabilization [62,63]. The CD spectra in the absence and in the presence of TB have similar shape, without any changes of peak positions, which indicates that the α-helical structure is still dominant. All these results reveal that the binding of TB could induce slight conformational changes in HSA, which is in good agreement with the previous results of 3D fluorescence spectroscopy.

### 2.9. Molecular Docking

The crystal structure of HSA (variant 1AO6) was used as the target for docking. Blind docking search resulted in finding one of the IBF binding sites (Arg209 and Glu354) and the warfarin binding site (Arg218 and Lys195). In further simulations we reduced the search space to a volume of size 30 × 30 × 30 Å centered on the warfarin binding site and the IBF binding sites.

**Figure 5:**
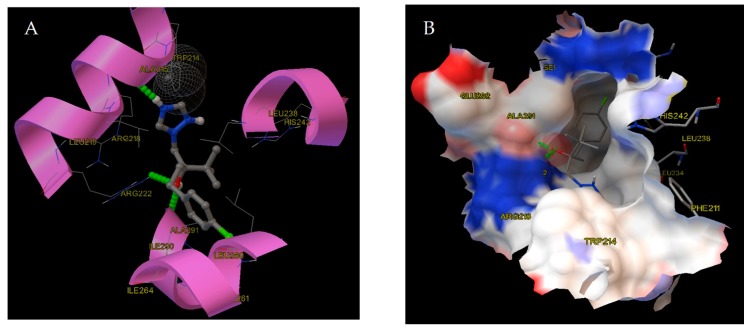
(**A**) Binding mode of TB to HSA. The secondary structure of the protein is shown and the neighboring amino acid residues are labeled. The hydrogen bonds are indicated by green lines. (**B**) Electrostatic potential of the ligand binding pocket in subdomain IIA (site I) of HSA. The negative and positive electrostatic potentials are colored red and blue, respectively.

The most stable binding in the warfarin cavity was docked with binding energy of −29.28 KJ/mol with error of computation being ±11.51 KJ/mol [64]. This is in agreement with the experimental measurements. The conformation is displayed in Figure 5A with its respective electrostatic potential in Figure 5B. The amino acids that are surrounding it are: Trp214, Ala215, Arg218, Leu219, Arg222, Leu238, His242, Leu260, Ala261, Ile-64, Ile290 and Ala291.

After further investigation of the electrostatic potential of the binding pocket, we deduced that the pocket is slightly positively charged and thus can interact with the negatively-charged apexes of TB (mainly the triazole ring, Cl^−^ locus and the hydroxyl group). For TB three hydrogen bonds were predicted from the conformation, as follows: the oxygen atom of the hydroxyl group receives the hydrogen from Arg222, the nitrogen atom of the triazole ring donates the hydrogen to the bond with Ala215, and the edge hydrogen of the hydroxyl group is donated to the bond with Ala291.

It is known that IBF has multiple HSA binding sites [65]. Because these interactions were outside of the scope of this study, we prioritized the two binding sites described in the protein 2BXG [66]. One is in the region of Arg209 and the other Tyr411. The simulations at these sites revealed the existence of stable binding modes with significant binding energies of −26.77 KJ/mol and −29.70 KJ/mol. This was, however, contrary to the results from the experiment where we did not observe any competition between binding of TB and IBF. We speculate that the reason for this is because these two specific sites have a region of highly negative electrostatic potential (Figure S9A,B) generated by the negatively charged residues Arg410, Lys413, Lys541 and Lys545 (Gly207, Arg209, Lys212, Lys323). The negatively charged Cl^−^ end of TB is unable to navigate these electrostatic fields. Unfortunately, AutoDockVina is evaluating individual conformations and not the possibility of reaching such conformations by taking some possible trajectory. Further study using molecular simulations could provide answers to this behavior.

In our simulation we also investigated various other known locations [67] like the Lys525, Lys199, Lys233, Cys34 and others. These simulations did not uncover any significant binding activity. From this point of view, we hypothesize that the main binding activity is happening at the warfarin binding site.

## 3. Materials and Methods

### 3.1. Chemicals and Reagents

TB (CAS Number 107534-96-3) was obtained from Sigma Aldrich, Darmstadt, Germany, with the purity ≥98.4%. TB was dissolved in spectroscopic grade 100% ethanol at concentration 10^−3^ mol/L. HSA (fatty acid-free, globulin-free, purity no less than 99%) was purchased from Sigma Aldrich, Darmstadt, Germany, and was used without further purification. A stock solution of HSA (concentration 5 × 10^−4^ mol/L) was prepared in Tris-HCl (0.05 mol/L Tris + 0.1 mol/L NaCl) buffer, pH 7.4 and conserved in the dark at 4 °C. Ketoprofen (KTF) and ibuprofen (IBF) were obtained from Sigma Aldrich, Darmstadt, Germany, with purity ≥98.0%. KTF and IBF were dissolved in 100% ethanol at the concentration 10^−3^ mol/L. Phosphate buffer (0.02 mol/L, pH 7.4) was used for DSC calorimetric and CD spectroscopic measurements. Tris(hydroxymethyl)aminomethane, NaCl, HCl, and other reagents were all of high purity. The buffers were prepared in the triple distilled water. All experiments were realized from 3 to 8 (fluorescence quenching and CD) times independently and presented data were summarized as the mean with the related standard deviation.

### 3.2. Differential Scanning Calorimetry

Differential scanning calorimetry measurements were performed on a Microcal VP-Capillary automated high-sensitivity differential calorimeter (Malvern Panalytical, Malvern, Worcestershire, UK) in the range of temperature from 25 to 95 °C at specific heating rate 1.5 °C/min. The concentration of HSA was fixed at 3 × 10^−5^mol/L. The concentration of TB was subsequently varied within the range 3 × 10^−5^–3 × 10^−4^ mol/L. DSC curves and chemical kinetic data were obtained using DSC thermoanalytical software (Version 8.0, OriginLab, Northampton, MA, USA).

### 3.3. Fluorescence Measurements

Fluorescence experiments were taken out with the RF 5301 PC spectrofluorimeter (Shimadzu, Kyoto, Japan) equipped with a 1 cm quartz cell. Excitation wavelength was set at 295 nm and fluorescence was collected in 300–500 nm emission wavelengths using 5nm/5nm slits. TB/HSA complexes for fluorescence spectroscopy were prepared by titration of TB into 2 × 10^−6^ mol/L HSA to achieve a final concentration of TB from 2 × 10^−6^ mol/L to 32 × 10^−6^ mol/L. After each titration the sample was 10 min stabilized. Decrease of the protein concentration by adding the TB in ethanol was minimal and did not reach 1.5%. All experiments were measured at three different temperatures, namely 25, 30 and 37 °C. The inner filter effect was eliminated by using very low concentration of HSA, which gives the absorbance less than 0.1 at excitation wavelength 295 nm.

Synchronous fluorescence spectra were recorded at room temperature. The interval of scanning between excitation and emission wavelength was fixed at 15 and 60 nm, respectively, and the emission was recorded from 200 to 400 nm.

Site markers competitive experiments were recorded at room temperature. The excitation wavelength was set at 295 nm and the emission was collect in the range 300–500 nm using 5nm/5nm slits. The concentrations of KTF, IBF and HSA were fixed at 2 × 10^−6^ mol/L. TB was then gradually added into KTF/HSA and IBF/HSA complexes. The 3D fluorescence spectra were also recorded at room temperature. The initial excitation wavelength was set at 220 nm with increments of 5 nm, and the emission was collected from 250 to 500 nm.

The different values of HSA concentrations were customized to the optimal conditions of the measurement methods used.

### 3.4. Circular Dichroism (CD) Studies

Far-UV CD spectroscopy experiments were recorded on a Jasco J-815 CD spectrometer (Jasco, Easton, MD, USA) equipped with a 0.1 cm quartz cell. at room temperature and with constant nitrogen flush. The CD spectra of HSA in the presence of TB were recorded from 190 to 270 nm and scan rate was 50 nm/min. Three scans were collected for each spectrum, taking the averages as the final data.

TB/HSA complexes for CD spectroscopy were prepared by titration of TB into 3 × 10^−6^ mol/L HSA to achieve a final concentration of TB from 3 × 10^−6^ mol/L to 1.5 × 10^−5^ mol/L. Tris-HCl buffer was exchanged for phosphate buffer (0.02 mol/L, pH 7.4) to eliminate the influence of Cl^−^ ions.

### 3.5. Molecular Docking

For our investigation we have used the HSA crystallographic data from the Brookhaven Protein Data Bank. The selected species was 1AO6 [67] as it describes HSA in its unbound state with sufficient precision. The molecular structure of TB was taken from PubChem database, entry 86102. For docking we used AutoDockVina [64]. We first performed blind docking for initial interaction site targeting. In the investigation we also included other binding sites that have been mentioned in literature [68]. Our main focus was on investigating TB activity in warfarin binding site.

## 4. Conclusions

Better understanding of the interaction of TB and other fungicides with various possible molecular and/or cellular targets is essential for the determination of their function in biological systems. The present paper focuses on results obtained in the study of TB associations with serum albumins represented by HSA.

In this study we investigated the interaction of triazole fungicide TB with HSA using calorimetric and spectroscopic methods, complemented with theoretical molecular modeling. As shown, TB interacts with HSA in vitro under simulated physiological conditions. The HSA fluorescence quenching by TB is, within the range of experimental concentration, attributed to a static mechanism. The thermodynamic parameters offer proof of structurally related binding modes, which are in good agreement with our molecular modeling results. The displacement of TB from HSA after addition of KTF indicated that Sudlow site I (subdomain IIA) was the probable binding site of TB to HSA. Furthermore, the binding of TB to HSA leads to slight conformational changes in HSA as corroborated by our 3D fluorescence and CD measurements outcomes.

Our results are also in good agreement with those of Zhang et al. [26], where slight binding interactions and small changes in the secondary structure of HSA after binding with triazole fungicides are reported.

## Figures and Tables

**Table 1:** Thermodynamic parameters of denaturing complexes of human serum albumin (HSA) and TB/HSA.

Ratio TB/HSA	t_D1_ (°C) *	t_D2_ (°C) ^+^	Δ*H* (kJ/mol)
0	57.20	65.53	527.97
1/1	57.86	67.08	604.75
5/1	59.67	71.57	652.22
7/1	60.46	72.93	710.02
10/1	60.49	72.99	730.66

* Denaturation temperature of the first transition. ^+^ Denaturation temperature of the second transition.

**Table 2:** Association constants and thermodynamic parameters for the interaction between HSA and TB.

Temperature (°C)	*K_SV_* (L/mol)	*K_A_* (L/mol)	*n*	Δ*G* (kJ/mol)	Δ*H* (kJ/mol)	Δ*S* (J/mol·K)
25 *	7.26 × 10^3^ ± 0.09	8.51 × 10^3^ ± 0.06	1.01 ± 0.02	−22.68 ± 0.001	−56.96 ± 0.02	−115.98 ± 0.07
30	4.77 × 10^3^ ± 0.02	4.85 × 10^3^ ± 0.02	0.98 ± 0.02	−22.10 ± 0.001		
37	3.15 × 10^3^ ± 0.02	3.46 × 10^3^ ± 0.09	0.96 ± 0.02	−20.99 ± 0.002		

* [28].

## Supplementary Materials

The Supplementary Materials are available online. Figure S1: DSC curves of HSA, Figure S2: Absorption spectra of TB, Figure S3: Hill plots of TB quenching effect on HSA fluorescence at the different temperatures, Figure S4: Van′t Hoff plot for the interaction between TB and HSA, Figure S5: Synchronous fluorescence spectra of HSA, Figure S6: Effect of site marker KTF to the TB/HSA system, Figure S7: Effect of site marker IBF to the TB/HSA system, Figure S8: CD spectra of HSA and TB/HAS, Figure S9: Electrostatic potential of the ligand binding pocket in site II, Table S1: Association constants of competitive experiments for the interaction between TB and HAS, Table S2: Peak position and intensity of fluorescence parameters for the interaction between HSA and TB.
