# Supplementary material for: Fungicide Tebuconazole Influences the Structure of Human Serum Albumin Molecule
Source: Molecules. 2019 Sep 2;24(17):3190. doi: 10.3390/molecules24173190 (PMC6749206; doi:10.3390/molecules24173190)
Supplement: Supplementary file 1 [file molecules-24-03190-s001.pdf]

molecules

Supplementary Material

Fungicide Tebuconazole Influences the Structure of Human Serum Albumin Molecule

Katarína Želonková [1], Samuel Havadej [1], ValériaVerebová [2], Beáta Holečková [3], Jozef Uličný [1] and Jana Staničová [2,4,*]

1 Faculty of Science, Pavol Jozef Šafárik University, Jesenná 5, 041 54 Košice, Slovakia; katarina.zelonkova@student.upjs.sk (K.Z.); samuel.havadej@student.upjs.sk (S.H.); jozef.ulicny@upjs.sk (J.U.)
2 Department of Chemistry, Biochemistry & Biophysics, University of Veterinary Medicine & Pharmacy, Komenského 73, 041 81 Košice, Slovakia; valeria.verebova@uvlf.sk (V.V.)
3 Department of Biology & Genetics, University of Veterinary Medicine & Pharmacy, Komenského 73, 041 81 Košice, Slovakia; beata.holeckova@uvlf.sk (B.H.)
4 First Faculty of Medicine, Charles University, Kateřinská 1, 121 08 Prague, Czech Republic
* Correspondence: jana.stanicova@uvlf.sk; Tel.: +421-915-984-613

Table S1. Peak position and intensity of fluorescence parameters for the interaction between HSA and TB.

Complex	Peak	Peak Position [$\lambda_{ex}/\lambda_{em}$ nm/nm]	Intensity of Fluorescence
HSA	a	250/250 → 310/310	221.58 → 830.90
	b	240/480	83.44
	1	275/340	674.46
	2	230/330	110.54
TB/HSA 16/1	a	250/250 → 310/310	1015.86 → 1015.88
	b	240/480	402.56
	1	275/340	628.73
	2	230/330	106.32

Table S2. Association constants of competitive experiments for the interaction between TB and SA.

Site Markers	K_A (L/mol)	n
Blank	$8.51 \times 10^3 \pm 0.09$	1.01 ± 0.02
KTF	$0.32 \times 10^3 \pm 0.09$	0.72 ± 0.02
IBF	$6.03 \times 10^3 \pm 0.09$	0.93 ± 0.02

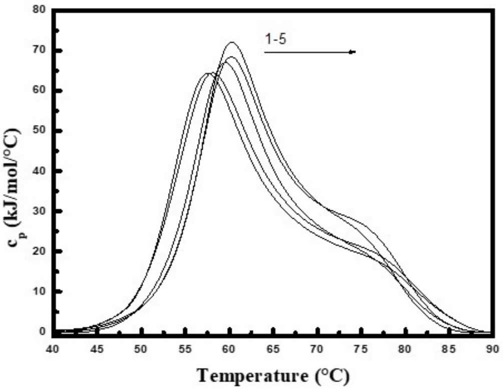

Figure S1. DSC curves of HSA: (**1**) DSC curve of HSA, c (HSA) = 3 × 10^{-5} mol/L; (**2–5**): TB/HSA, 3 × 10^{-5}mol/L HSA in the presence of 3 × 10^{-5}; 15·10^{-5}; 21 × 10^{-5}; 3 × 10^{-4}mol/L TB.

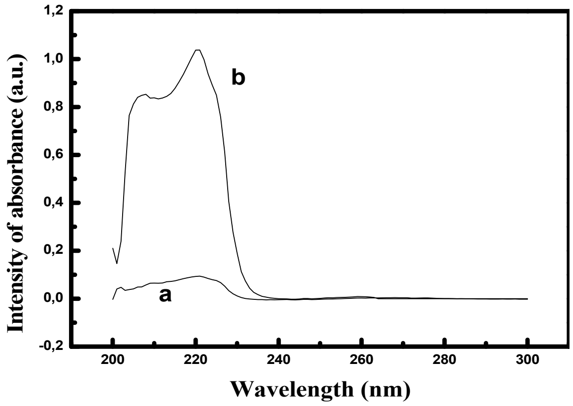

Figure S2. Absorption spectra of TB. (**a**) c(TB) = 2 × 10^{-6} mol/L; (**b**) c(TB) = 32 × 10^{-6} mol/L; pH = 7.4; t = 25 °C.

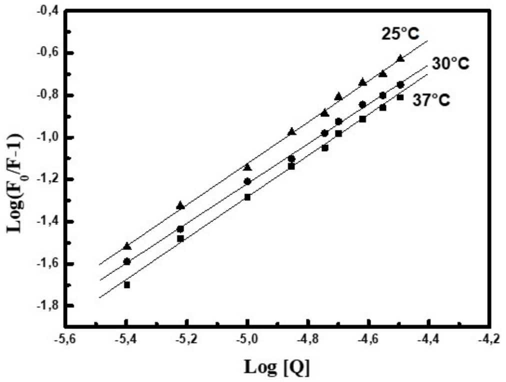

Figure S3. Hill plots of TB quenching effect on HSA fluorescence at the different temperatures. c (HSA) = 2 × 10^{-6}mol/L; c (TB) = 0–32 × 10^{-6}mol/L; λ_{exc} = 295 nm; λ_{em} = 300–500 nm; pH = 7.4; t = 25, 30 and 37 °C.

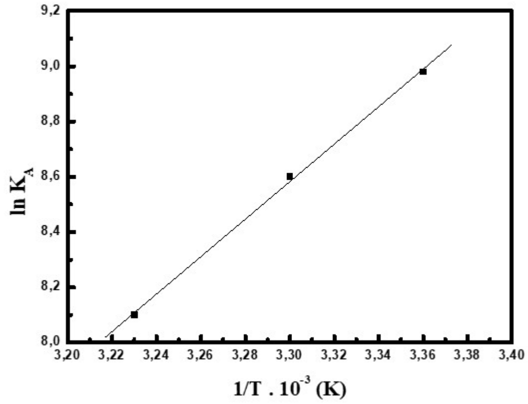

Figure S4. Van't Hoff plot for the interaction between TB and HSA. c (HSA) = 2 × 10^{-6} mol/L; c (TB) = 0–32 × 10^{-6} mol/L; λ_{exc} = 295 nm; λ_{em} = 300–500 nm; pH = 7.4.

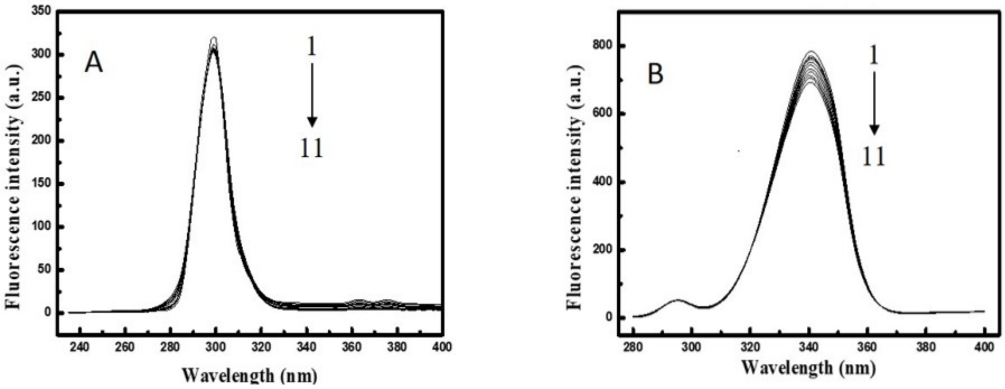

Figure S5. (**A**) Synchronous fluorescence spectra of HSA in the presence of different concentrations of TB; c (HSA) = 2 × 10^{-6} mol/L; c (TB) = 0–32 × 10^{-6} mol/L; $\Delta\lambda$ = 15 nm. (**B**) Synchronous fluorescence spectra of HSA in the presence of different concentrations of TB; c (HSA) = 2 × 10^{-6} mol/L; c (TB) = 0–32 × 10^{-6} mol/L; $\Delta\lambda$ = 60 nm.

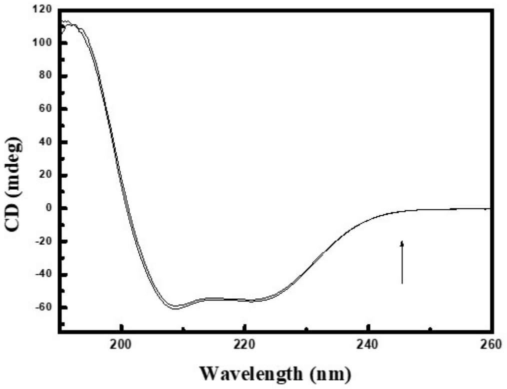

Figure S6. CD spectra of HSA and TB/HSA; c (HSA) = 3 × 10^{-6} mol/L; c (TB) = 15 × 10^{-6} mol/L; pH = 7.4; t = 25 °C.

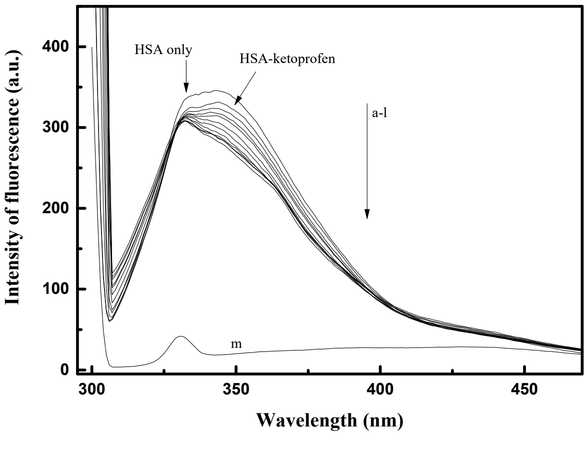

Figure S7. Effect of site marker ketoprofen to the TB/HSA system; c (KTF) = c (HSA) = 2 × 10^{-6} mol/L; c (TB) = 0–32 × 10^{-6} mol/L; (**a–l**): TB/HSA: 0,2,4,6,10,14,18,20,24,28,32; (**m**): fluorescence spectrum of ketoprofen, c (KTF) = 2 × 10^{-6} mol/L; pH = 7.4; t = 25 °C.

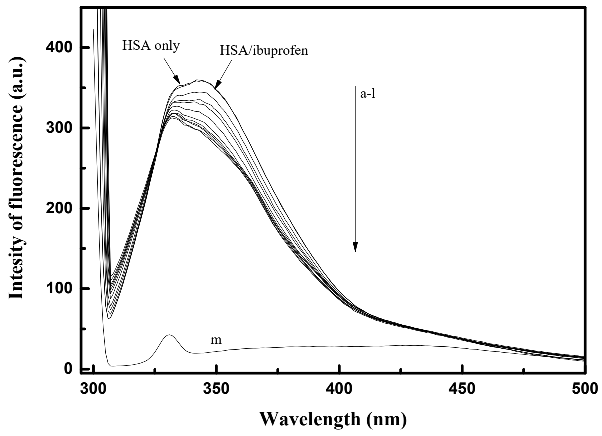

Figure S8. Effect of site marker ibuprofen to the TB/HSA system; c (IBF) = c (HSA) = 2 × 10^{-6} mol/L; c (TB) = 0–32 × 10^{-6} mol/L; (**a–l**): TB/HSA: 0,2,4,6,10,14,18,20,24,28,32; (**m**): fluorescence spectrum of ibuprofen, c (IBF) = 2 × 10^{-6} mol/L; pH = 7.4; t = 25 °C.

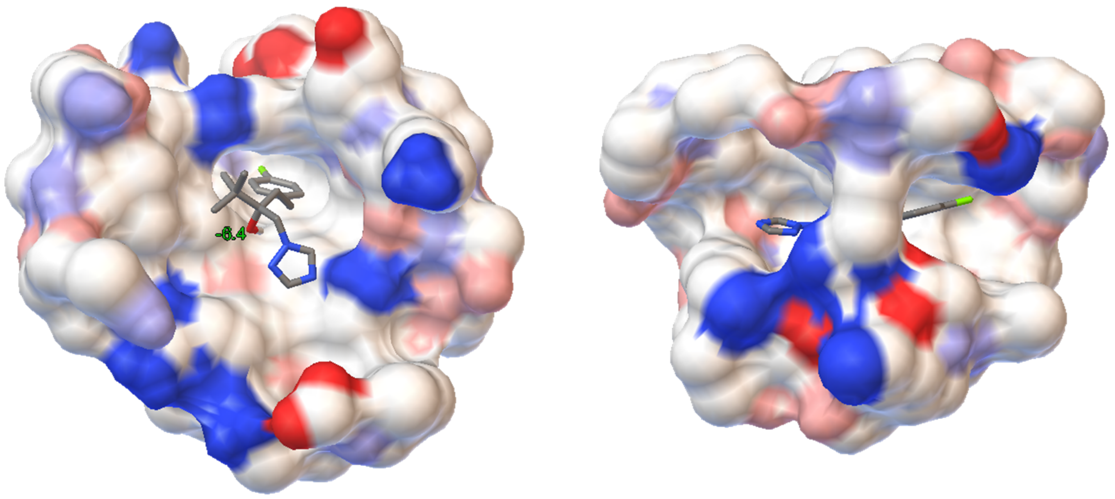

A B

Figure S9. Electrostatic potential of the ligand binding pocket in subdomain IIIA (site II) of HSA. The negative and positive electrostatic potentials are colored red and blue, respectively.
